# Clinical Impact of Genomic Information in Pediatric Leukemia

**DOI:** 10.3389/fped.2017.00263

**Published:** 2017-12-14

**Authors:** Emilie Lalonde, Gerald Wertheim, Marilyn M. Li

**Affiliations:** ^1^Department of Genetics, Perelman School of Medicine, University of Pennsylvania, Philadelphia, PA, United States; ^2^Department of Pathology and Laboratory Medicine, Perelman School of Medicine, University of Pennsylvania, Philadelphia, PA, United States; ^3^Center for Childhood Cancer Research, The Children’s Hospital of Philadelphia, Perelman School of Medicine, University of Pennsylvania, Philadelphia, PA, United States

**Keywords:** genomic profiling, pediatric leukemia, diagnosis, prognosis, therapy

## Abstract

Pediatric leukemia remains a significant contributor to childhood lethality rates. However, recent development of new technologies including next-generation sequencing (NGS) has increased our understanding of the biological and genetic underpinnings of leukemia, resulting in novel diagnostic and treatment paradigms. The most prevalent pediatric leukemias include B-cell acute lymphoblastic leukemia (B-ALL) and acute myeloid leukemia (AML). These leukemias are highly heterogeneous, both clinically and genetically. There are multiple genetic subgroups defined by the World Health Organization, each with distinct clinical management. Clinical laboratories have started adopting genomic testing strategies to include high-throughput sequencing assays which, together with conventional cytogenetic techniques, enable optimal patient care. This review summarizes genetic and genomic techniques used in clinical laboratories to support management of pediatric leukemia, highlighting technical, biological, and clinical advances. We illustrate clinical utilities of comprehensive genomic evaluation of leukemia genomes through clinical case examples, which includes the interrogations of hundreds of genes and multiple mutation mechanisms using NGS technologies. Finally, we provide a future perspective on clinical genomics and precision medicine.

## Introduction

Leukemia accounts for 30% of all childhood malignancies in the United States (1, 2). Advances in the biological understanding of leukemogenesis and improved treatment options have significantly increased survival rates, with childhood mortality rates decreasing nearly fourfold from 1975 to 2016 (1–3). Pediatric leukemia is both clinically and genetically heterogeneous, and identifying personalized management schemes for every child with leukemia requires a thorough molecular investigation (4). Empowered by the development of next-generation sequencing (NGS) technologies, a large number of leukemia-associated genetic alterations have been elucidated in recent years. The wealth of basic research illustrating the genetic heterogeneity in pediatric leukemia has led to management changes in the workup of patients with leukemia and involves novel technologies. Many recent studies support the use of comprehensive genomic characterization of pediatric cancers in identifying potentially actionable mutations (4, 5).

This article focuses on the genomic evaluation of pediatric leukemia that may enable precision medicine. Biological and technical perspectives are considered in this review as we discuss molecular genetic strategies required for thorough analysis of these genetically heterogeneous malignancies. We discuss the strengths and weaknesses of various technologies required to inform clinical diagnosis, prognosis and management of pediatric leukemia, with an emphasis on the testing strategy employed at the Division of Genome Diagnostics from the Children’s Hospital of Philadelphia (CHOP).

## Molecular Strategies for Genetic Testing in Leukemia

The repertoire of clinically significant genomic mutations in hematological malignancies includes a wide variety of alterations. These include single-nucleotide variants (SNVs) leading to missense or nonsense amino acid changes; splice site substitutions affecting normal RNA processing; small deletions, duplications, insertions, or a combination of these commonly referred to as indels; copy number variations (CNVs); and large structural variations, disrupting the function of the genes involved or resulting in new fusion genes. Many of these alterations are critical for clinical diagnosis, prognosis, and therapy of patients with leukemia. Various molecular biology techniques are used clinically to detect these alterations. Chromosomal analysis, fluorescent *in situ* hybridization (FISH), and targeted Sanger sequencing have been the primary tools of detecting these alterations and remain part of the standard care (6). In recent years, high-throughput molecular technologies, including chromosomal microarray analysis (CMA) and NGS, have enhanced the capability to characterize critical genomic variations, and a combination of both classic and novel molecular technologies are used to assess clinically relevant mutations.

### Conventional and Molecular Cytogenetics

Cancer cytogenetics started with the identification of the so-called Philadelphia chromosome, caused by a translocation between chromosomes 9 and 22, in the majority of patients with chronic myelocytic leukemia (CML) (7, 8). Numerous additional chromosomal rearrangements and copy number changes have since been identified. These abnormalities are either pathognomonic for specific hematologic malignancies, or convey prognostic and therapeutic implications. However, the genomic resolution is low (approximately 20 Mb), and viable, dividing cells must be obtained. FISH technology allows the detection of small (50–100 kb) deletions/duplications/amplifications and gene fusions, and permits the direct visualization of these alterations in interphase cells, yet is restricted to specific sequences of probes. As a complement to chromosomal analysis, CMA enables high-resolution detection of CNVs, allelic imbalance, and loss of heterozygosity (LOH) (9, 10), but cannot detect balanced structural changes, such as translocations or inversions. Despite the drawbacks of being manually intensive and not being scalable, karyotyping and FISH are still routinely performed for all diagnostic specimens as part of standard care.

### Polymerase Chain Reaction (PCR) and Sanger Sequencing

Polymerase chain reaction followed by fragment analysis or Sanger sequencing can be used to detect clinically relevant alterations in leukemia, such as *FLT3*-ITD, B- and T-cell gene rearrangement analyses, or *JAK2, NPM1*, and many other gene mutations (11–15). Quantitative reverse transcription PCR (RT-qPCR) or digital droplet PCR have exquisite analytical sensitivity and may thus be used for detection of minimal residual diseases (MRDs) and for monitoring disease burden (e.g., *BCR-ABL1* fusion transcript levels in CML during tyrosine kinase inhibitor treatment) (16). As with FISH, however, these technologies are limited in the number of mutations they can evaluate at the same time, and are thus time consuming and costly.

### Next-Generation Sequencing

As the cost of NGS decreases and the knowledge of mutational landscapes involved in leukemogenesis increases, NGS-based tests are quickly assuming critical roles in clinical cancer care. NGS is capable of detecting all forms of genomic alterations, and can scale from targeted panels (typically 50 to a few 100 genes), to whole-exome sequencing (WES) covering 1% of the genome, to whole-genome sequencing (WGS) (17). WES evaluates all coding sequences of the genome and WGS offers the most comprehensive mutational analysis by sequencing the whole genome (Table 1). However, both WES and WGS are not routinely used in clinical laboratories due to the cost, long turnaround time, and extensive efforts required for data analysis at present time (18, 19). As such, clinical laboratories have begun to adopt NGS panel assays restricted to detecting mutations in specific genes known to be essential in leukemia. Compared to traditional Sanger sequencing or FISH assays, targeted NGS panel tests are far more robust, cost-effective, and can provide more comprehensive genomic information in a shorter time-frame (19–21). For these targeted assays, different strategies have been developed to capture the regions of interest from both DNA and RNA, including amplicon sequencing from multiplex PCR, hybrid capture using magnetic beads, and anchored multiplex PCR. Whereas multiplex PCR may be best suited for small panel sizes and has lower input requirements, hybrid capture can target hundreds of genes and results in more even coverage which enables copy number analysis. Anchored multiplex PCR is best suited to identify structural rearrangements leading to known or novel fusion genes. However, it is expected that broader assays such as exome, genome, and transcriptome sequencing, will eventually replace panel-based tests as costs continue to decrease and analytical methods improve.

**Table 1:** Comparison of genomic technologies commonly used in clinical laboratories for leukemia profiling.

	Cytogenetics	Fluorescent in situ hybridization	Chromosomal microarray analysis	Sanger sequencing	Next-generation sequencing
Resolution	10–20 Mb	50–100 kb	1–100 kb	1 bp	1 bp

Sample type	Fresh tissue: PB, BM, tumor	PB, BM, fresh, FF, FFPE, etc.	PB, BM, fresh, FF, FFPE, etc.	PB, BM, fresh, FF, FFPE, etc.	PB, BM, fresh, FF, FFPE, etc.

Aberration detection	Del/Dup/Amp, insertion, translocation	Del/Dup/Amp, translocation	Del/Dup/Amp	SNVs, indels	Del/Dup/Amp, translocation, SNV, indel, fusions, SV

Loss of heterozygosity	No	No	Yes^a^	No	Yes

Qualitative or quantitative	Qualitative with low sensitivity	Quantitative	Semi quantitative	Qualitative with relatively low sensitivity	Quantitative with high sensitivity

Genomic coverage	Whole genome	Targeted	Whole genome	Targeted	Targeted/whole genome

Scalability	No	No	Yes	No	Yes

*^a^Only arrays containing SNPs*.

*PB, peripheral blood; BM, bone marrow; FF, fresh frozen; FFPE, formalin fixed paraffin imbedded; del, deletion; dup, duplication; Amp, amplification; SV, structural variation; UPD, uniparental disomy; SNVs, single nucleotide variants*.

In our clinical laboratory, we have developed a NGS-based comprehensive hematological cancer panel to profile genomic alterations of all leukemia patients at diagnosis and at relapse. This panel is designed to interrogate SNVs, indels, CNVs/LOH in more than 100 genes, and both known and novel fusions associated with 106 major fusion partners. Variants identified from these assays are put into clinical context using the standards and guidelines for somatic variant interpretation and reporting defined by AMP, ASCO, CAP, and ACMG, highlighting clinically actionable variants (22). Although this thorough approach has required extensive investment in infrastructure, equipment, information technology, data storage, and skilled personnel, the enhanced analysis has clearly impacted care of pediatric leukemia patients.

## Clinical Utility of Genomic Analysis in Leukemia

### Genomic-Guided Management in Acute Lymphoblastic Leukemia (ALL)

Acute lymphoblastic leukemia is the most common childhood malignancy accounting for 75–80% of pediatric leukemia and 26% of cancer diagnosed before 14 years of age (23, 24). Genetic rearrangements and CNVs characterize 10 different B-cell ALL subgroups as defined by the World Health Organization (WHO), including two provisional groups, each with distinct clinical considerations (25). Recent guidelines from the College of American Pathologists and the American Society of Hematology echo the WHO scheme in terms of the importance of genetic testing in acute leukemias (26). These recommendations include testing for common genomic structural changes and recurrent point mutations commonly seen in pediatric ALL and acute myeloid leukemia (AML).

The first provisional WHO subgroup, Ph-like B-cell acute lymphoblastic leukemia (B-ALL), is defined by a similar gene expression profile to B-ALL with the Philadelphia chromosome yet lacks a *BCR-ABL1* fusion (27). Approximately 10% of pediatric B-ALL may be classified as Ph-like ALL, and show poor response to induction chemotherapy (28). These leukemias harbor gene fusions involving tyrosine kinases including *ABL1* (not associated with *BCR*), *ABL2, PDGFRB, NTRK3, TYK2, CSF1R*, and *JAK2*, rearrangements involving cytokine receptor-like factor 2 (*CRLF2*), or alterations associated with erythropoietin receptor gene. These mutations lead to activation of growth promoting kinases or of cytokine signaling pathways (29). ALL with *CRLF2* rearrangements often found in combination with *JAK* mutations, resulting in activation of JAK–STAT pathways (30, 31). These distinct alterations may render these leukemias sensitive to tyrosine kinase inhibitors such as dasatinib, JAK inhibitors such as ruxolitinib, or other small molecule inhibitors (29). Current clinical trials, such as COG AALL1521, incorporate mutational testing and small molecular inhibitors in their protocols (https://clinicaltrials.gov/, e.g., NCT02723994). It is, therefore, imperative to include Ph-like alterations in the molecular testing of B-ALL. Additionally, many of the kinases involved in fusions have multiple fusion partners with distinct downstream biological effects, requiring testing modalities capable of detecting multiple known and even novel fusion partners.

**Figure 1:**
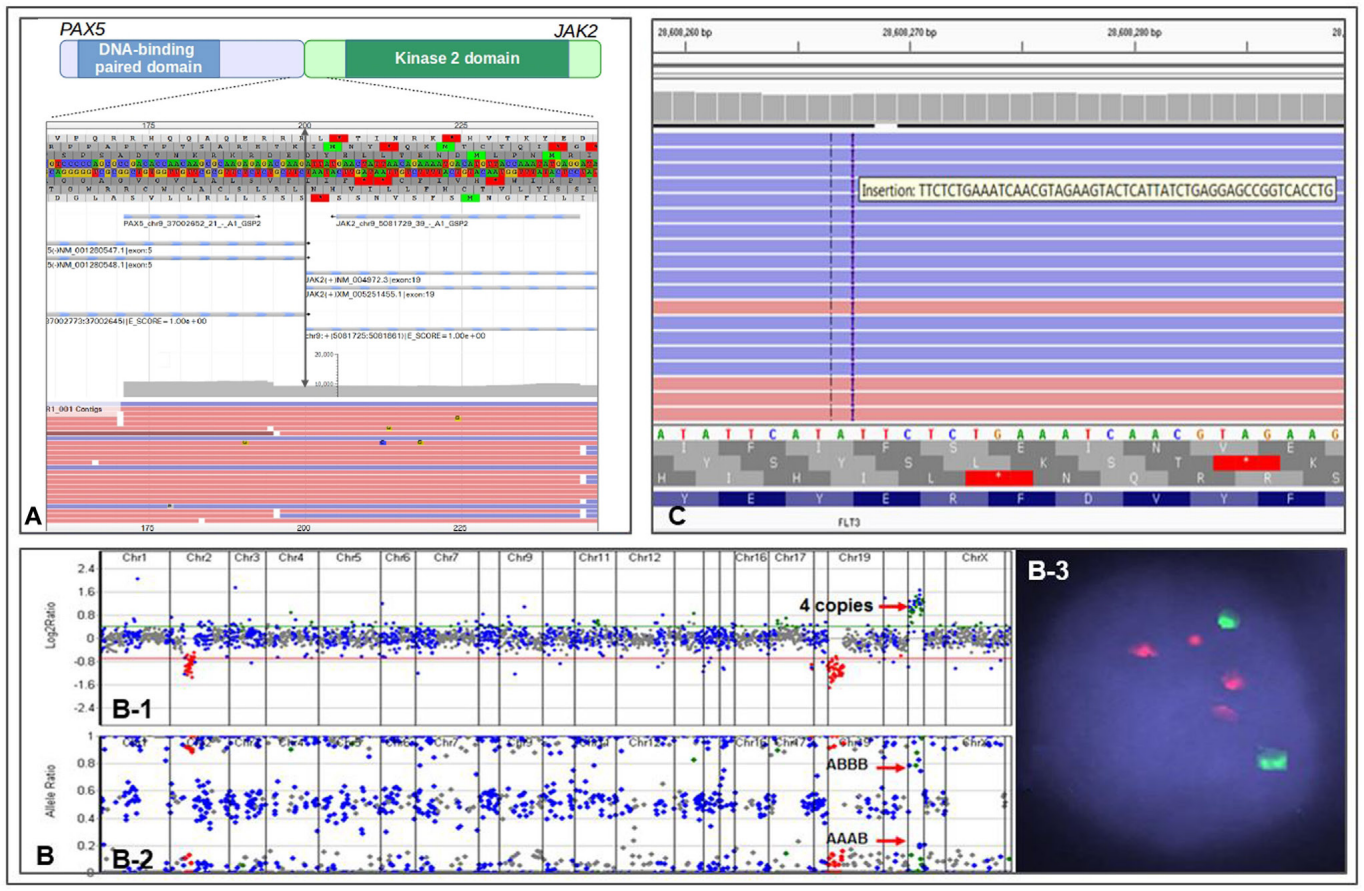
Representations of different genomic alterations identified by CHOP Comprehensive Hematological Cancer Panel. **(A)** a PAX2–JAK2 fusion detected in an 11-month-old patient with residual/recurrent B-cell lymphoblastic leukemia. Double arrowed line indicates exon 5 of PAX2 is fused to exon 19 of JAK2, gray area indicates reading depth, red and blue horizontal bars are representative reads. **(B)** Copy number analysis using next-generation sequencing data from a patient with iAMP ALL. (B-1) Copy number variations analysis based on reading depth; red arrow indicates four copies of RUNX1 genomic region. (B-2) B-allele frequency analysis demonstrating SNP separation due to triplication of one allele; red arrows indicate genotype information of AAAB and ABBB (the genotype would be AABB if it were duplication of both allele). (B-3) FISH showing two ETV6 signals (green) and 4 RUNX1 signals (red). **(C)** IGV view showing a FLT3 ITD in an 11-year-old patient with acute myeloid leukemia.

The NGS testing repertoire in our laboratory incorporates novel fusion kinases involved in Ph-like B-ALL. We recently performed testing on an 11-year-old patient with relapsed B-ALL. The patient had previously tested negative by conventional technologies; however, we identified a *PAX5–JAK2* fusion (Figure 1A) along with biallelic loss of TP53 function. *PAX5* is a master regulator of B-cell development, and the chimeric protein maintains both the PAX5 DNA-binding activity and the JAK2 kinase activity (32). Both loci are promiscuous in translocation partners, as *JAK2* can include up to 14 different 5′ partners in Ph-like B-ALL (30), and *PAX5* has at least 12 fusion partners (33). The *PAX5–JAK2* fusion has previously been identified in Ph-like B-ALL, and the chimeric protein has shown to be sensitive to JAK2 inhibitors by *in vitro* studies (32). The identification of the *PAX5–JAK2* fusion indicates that the patient has a Ph-like ALL with poor prognosis and opens the door for possible therapy with JAK inhibitors.

The other provisional B-ALL subtype is characterized by intra-chromosomal amplification of chromosome 21 (i.e., iAMP21) caused by breakage-fusion-bridge cycles followed by chromothripsis or other complex rearrangements (25, 34). The entity is defined as at least three copies of the *RUNX1* gene on one chromosome 21, although the region of amplification is often larger, and may be associated with subtelomeric deletions of chromosome 21 (31, 34). It is mostly found in older children and is also associated with very poor prognosis with standard treatment protocols (34). Nevertheless, multiple clinical trials have demonstrated that more intensive chemotherapy regimens geared toward high-risk patients are effective in these patients, and counteract the adverse prognostic impact of iAMP21 (35, 36).

One of the major benefits of using a NGS-based genetic testing strategy is the ability to detect multiple types of aberrations in a single assay. Indeed, we recently identified a case of iAMP21 ALL using our Comprehensive Hematological Cancer Panel. Our NGS analysis software uses sequencing depth and common SNPs in the targeted regions to assess copy number status and genotype information at the same time. We identified a patient with B-ALL who showed four *RUNX1* signals by interphase FISH. This pattern could represent two signals on each chromosome 21, or at least three signals on a single chromosome, the latter of which indicates an iAMP21. NGS analysis showed four copies of *RUNX1* gene and a clear “AAAB” and “ABBB” heterozygous SNP pattern in the *RUNX1* chromosome region, demonstrating an iAMP21 (Figure 1B). The patient was immediately placed on an appropriate high-risk chemotherapy regimen.

### Genomic-Guided Prognostication in AML

Acute myeloid leukemia is an aggressive leukemia, with a cure rate of only approximately 60% for pediatric patients (37). Patients were historically classified into distinct subtypes of AML based on cell morphology and cytochemistry, yet are currently defined mainly by genetic abnormalities (38, 39). In 2001, the WHO classification of the myeloid neoplasms first recognized three subtypes of AML with recurrent genetic abnormalities (39). In 2008, the WHO classification included nine genomic alteration-based AML subtypes (40). The 2016 updates recognizes 11 genetically defined AML subtypes (25), highlighting the clinical significance of genomic testing in the care of AML patients.

A recent experience at CHOP illustrates well the importance of integrated genomic workup for AML. An 11-year-old patient with cytogenetically normal AML (CN-AML) was referred for further genetic testing for risk stratification. The Comprehensive Hematological Cancer Panel identified a *FLT3* internal tandem duplication (*FLT3* ITD) (Figure 1C) along with two other somatic variants, *IDH2* p.R140Q and *GATA2* p.W10*. *FLT3* ITD is often missed by NGS-based assays due to the size of the duplications. However, we have designed our panel and bioinformatics analysis pipeline to enable the detection of this mutation. The *FLT3* variant changes the patient’s prognosis from intermediate (based on CN-AML) to poor (25, 41–43). Moreover, recent clinical trials suggest adding FLT3 inhibitor to frontline chemotherapy in *FLT3*-mutated AML confers a survival benefit (44). The *FLT3* ITD present in this patient qualifies her for clinical trials investigating *FLT3* ITD targeted therapies.

### Genomic-Guided Diagnosis: Identifying RAS-Pathway Mutations in JMML

Juvenile myelomonocytic leukemia (JMML) is a rare type of leukemia found mainly in children less than 2 years of age (45). The prognosis of JMML is poor, and without treatment it is most often fatal within 5 years of life. Hematopoietic stem cell transplant is currently the only curative approach for JMML. Since both the clinical presentation and the morphologic features of JMML can resemble reactive processes, molecular studies are usually required for diagnosis. Most individuals with JMML have mutations in the RAS pathway, particularly *PTPN11, KRAS, NRAS, CBL*, or *NF1*. Some mutations in the RAS-MAPK pathway genes can occur in the germline and these patients are at risk for JMML-like myeloproliferations. JMML patients without RAS mutations may have mutations in *ASXL1, SETBP1, RUNX1, JAK3*, and *SH2B3* (46, 47), all of which have also been observed as secondary mutations in RAS-mediated JMML (46). In the 2016 WHO classification, the RAS-pathway mutations are among the diagnostic criteria for JMML (25).

Our Comprehensive Hematologic Cancer Panel includes the RAS-pathway genes and allows for rapid diagnosis of JMML. This is illustrated by the case of a 19-month-old boy with splenomegaly and WBC of 50.7 million per microliter with high count of monocytes, an absolute monocytosis, and thrombocytopenia. Blasts in both the peripheral blood and bone marrow were <20%. A rapid FISH analysis was negative for a *BCR–ABL1* translocation. The NGS panel was completed within 5 days of sample acquisition, and identified a missense mutation in *PTPN11* (c.181G>dT, p.Asp61Tyr). The genomic information helped us make the diagnosis within one week which allowed physicians to construct appropriate therapeutic strategies in a timely manner. Sanger sequencing on cultured and direct skin biopsies did not detect the variant, confirming the somatic origin.

### Genomic-Guided Disease Monitoring

The ability to detect a large variety of mutations allows the potential for tracking disease burden and relapse through molecular assays. MRD detection can be used to assess therapeutic response and is invaluable for patient management (14, 48–50). Historically, MRD tracking is done by using flow cytometry or by RT-qPCR assays to detect specific aberrations such as *BCR–ABL1* fusion (48). In CML, an International Scale has been established to allow standardization and comparison of results from RT-qPCR *BCR–ABL1* assays from different laboratories, and validated clinical endpoints have been established (51, 52). With the discovery of additional recurrent genetic mutations in hematological malignancies, new RT-qPCR assays have been developed to provide molecular tracking for MRD in a larger proportion of patients (53). This approach is particularly useful for patients undergoing targeted therapy.

**Figure 2:**
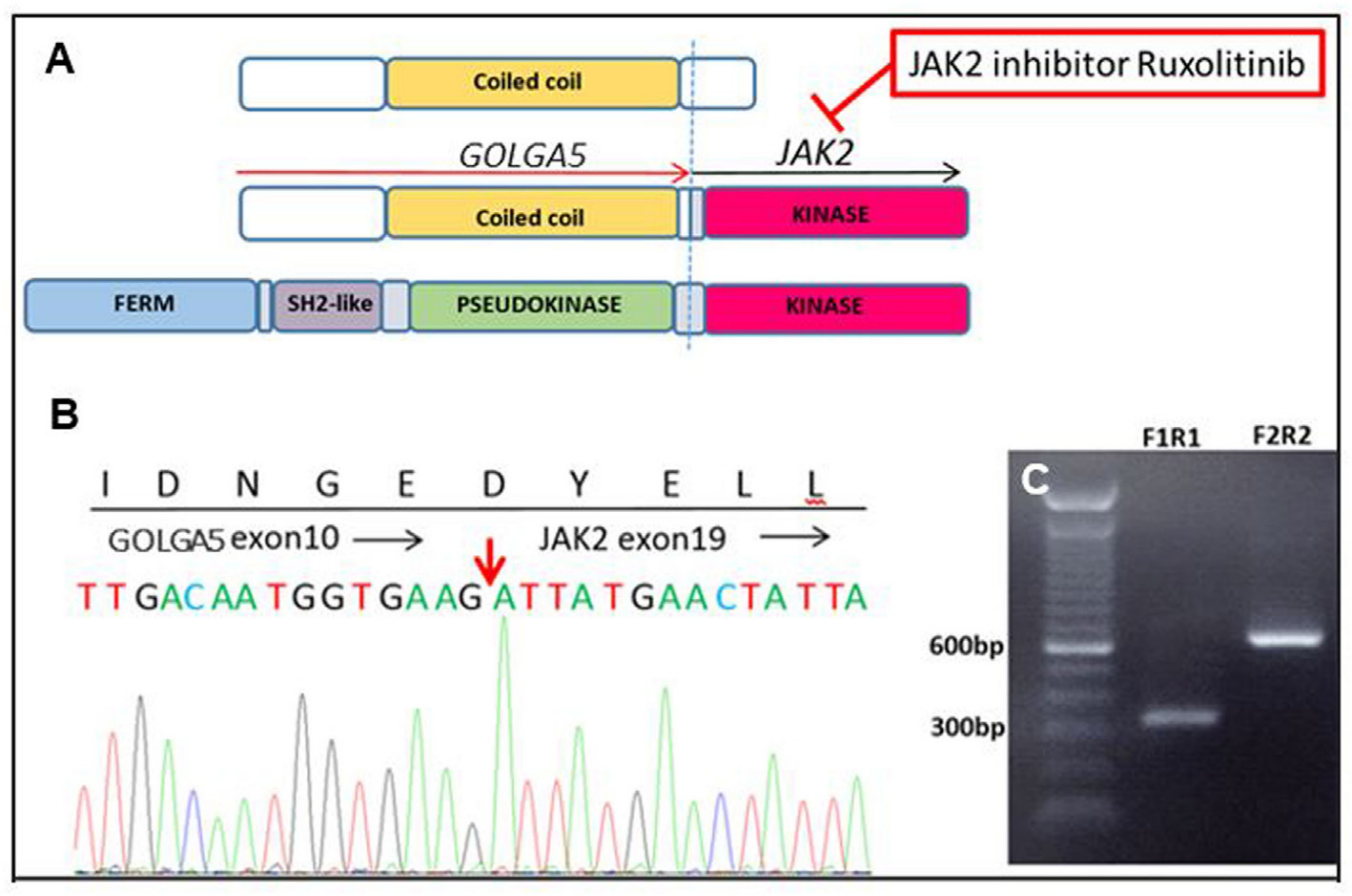
A novel GOLGA5–JAK2 fusion was identified in an 11-year-old boy with very high-risk acute lymphoblastic leukemia. **(A)** Schematic representation of protein domains of the GOLGA5–JAK2 fusion protein. **(B)** Sanger confirmation of the GOLGA5–JAK2 fusion. The red arrow indicates the breakpoint of the fusion transcript. **(C)** Gel electrophoresis of nested polymerase chain reaction (PCR) products from the diagnostic bone marrow sample; F1R1—PCR product of inner forward and reverse primers; F2R2—PCR product of outer forward and reverse primers.

Genomic analysis on a diagnostic bone marrow from an 11-year-old boy with very high-risk ALL and induction failure showed a novel *GOLGA5–JAK2* fusion, indicating a Ph-like B-ALL (Figure 2). Upon detection of the novel fusion, JAK inhibitor ruxolitinib was added to the therapy and a custom RT-qPCR assay was designed specifically for this patient to monitor the JAK2 fusion transcript levels during therapy. A nearly two-log reduction in the amount of JAK2 fusion transcripts was observed one month after the ruxolitinib treatment initiation, and the fusion transcripts were undetectable after 5 months of treatment. The patient was then enrolled in a clinical trial to receive CAR-T immunotherapy followed by matched sibling donor bone marrow transplant (BMT) and has been in remission ever since.

## Summary and Perspectives

The technical advances in genomic research and their clinical applications have resulted in tremendous clinical improvements within the last decade. In hematological cancers, in particular, molecular profiling is used to diagnose disease, stratify risk, guide therapy, and monitor treatment responses with increasing accuracy (54). Channeling the power of artificial intelligence, several large initiatives have begun acquiring, storing, and retrospectively mining genomic and clinical information to inform treatment decisions for new patients (48–50). This knowledge bank approach calculates an individual patient’s risk profile for various treatment options, based on observations from prior patients. Gerstrung and colleagues recently illustrated that individually tailored management decisions based on this approach could decrease the number of hematopoietic cell transplants in AML patients by 20–25% while maintaining the same overall survival rates (51).

Targeted NGS panels currently have a clinical advantage over exome and genome sequencing, due to faster turnaround times and higher sequencing depths resulting in higher analytical sensitivity and specificity (52). However, using targeted panels introduces significant logistical issues, in particular the need to re-design, re-optimize, and re-validate panels as new targets are discovered and become clinically relevant, imposing a burden on clinical laboratories wishing to update panel content on a regular basis. Targeted panels are also at risk of missing rare novel genomic alterations that are not included in a specific panel and are not suitable for many structural variations due to its reduced complexity. Given the decreasing cost and continued evolution of sequencing technologies and analytical methodologies, WGS, with its ability to identify SNVs, indels, CNVs/LOH, and structural variants simultaneously, and whole transcriptome sequencing that evaluates gene expression profiles, may soon supersede targeted panels (29, 53). Yet even WGS has limitations in that 8% of the genome cannot be sequenced due to repetitive sequences (55). Novel strategies such as long-read, single-molecule sequencing (e.g., PacBio, Oxford Nanopore) can fill some of these coverage gaps. These methods also do not require target amplification thereby reducing read strand bias (55–59). Linked read sequencing (10× Genomics), may also capture repetitive sequence regions of the genome, and enable strand phasing by using a barcoded, microfluidics approach (60). Development of these and other new technologies along with more sophisticated bioinformatics tools will permit an even more complete clinical representation of the molecular alterations in leukemia and offer patients exquisitely precise diagnosis, prognosis, and therapeutic opportunities.

## Author Contributions

EL, GW, and ML wrote, edited, and approved the final manuscript.

## Conflict of Interest Statement

The authors declare that the research was conducted in the absence of any commercial or financial relationships that could be construed as a potential conflict of interest.
